# Supplementary material for: Approaches Used to Describe, Measure, and Analyze Place of Practice in Dentistry, Medical, Nursing, and Allied Health Rural Graduate Workforce Research in Australia: A Systematic Scoping Review
Source: Int J Environ Res Public Health. 2022 Jan 27;19(3):1438. doi: 10.3390/ijerph19031438 (PMC8834932; doi:10.3390/ijerph19031438)
Supplement: Supplementary file 1 [file ijerph-19-01438-s001.zip › Supplementary material2.pdf]

Supplementary Material S2. Electronic searches

Database	Citations retrieved
Ovid MEDLINE	678
CINAHL (EBSCOhost)	619
PsycInfo (EBSCOhost)	192
Embase (Elsevier)	858
Total	2347
Duplicate papers	1218
Total left to screen	1129

Database searches were conducted on the 3 September 2021.

Database(s): Ovid MEDLINE(R) Epub Ahead of Print, In-Process & Other Non-Indexed Citations, Ovid MEDLINE(R) Daily, Ovid MEDLINE and Versions(R) .

Search strategy:.

Query	Search terms
1	exp Nursing/ or exp Students, Nursing/
2	(nurs* or nurs* students or midwife* or midwife* students).kf,tw.
3	exp Health Occupations/
4	exp Health Workforce/ or exp Allied Health Personnel/
5	exp Students, Medical/ or exp Physicians/ or exp Dentists/
6	(medic* or doctor* or medic* students or dentist*).kf,tw.
7	(physiotherap* or speech path* or dietitian* or occupational therap* or podiatr* or social work* or psych* or pharm* or chiropractor* or audiolog* or allied health students or exercise physio* or medical imag*).kf,tw
8	1 or 2 or 3 or 4 or 5 or 6 or 7
9	(student* or graduat*).kf,tw.
10	exp Rural Health/ or exp Rural/ or exp Rural Population/ or exp Rural Health Services/
11	(rural* or remote* or region*).kf,tw.
12	10 or 11
13	exp Australia/
14	(Australia* or Tasmania* or Victoria* or New South Wales or Queensland or Northern Territor* or Western Australia* or South Australia*).kf,tw.
15	13 or 14
16	8 and 9 and 12 and 15
17	Limit 16 to yr=2010-Current

Database(s): CINAHL (EBSCOhost).

Search strategy:.

Query	Search terms
S1	(MH “Nursing+”) OR (MH "Students, Nursing"+)
S2	TI (nurs* or nurs* students or midwife* or midwife* students) OR AB (nurs* or nurs* students or midwife* or midwife* students)
S3	(MH "Health Occupations"+) OR (MH “Allied Health Personnel”+) OR (MH “Health Workforce”+)
S4	(MH "Students, Medical"+) OR (MH “Physicians”+) OR (MH “Dentists”+)
S5	TI (medic* or doctor or medical students or doctor students or dentist*) OR AB (medic* or doctor or medical students or doctor students or dentist*)

S6	TI (physiotherap* or speech path* or dietitian* or occupational therap* or podiatr* or social work* or psych* or pharm* or chiropractor* or audiolog* or allied health students or exercise physio* or medical imag*) OR AB (physiotherap* or speech path* or dietitian* or occupational therap* or podiatr* or social work* or psych* or pharm* or chiropractor* or audiolog* or allied health students or exercise physio* or medical imag)
S7	S1 OR S2 OR S3 OR S4 OR S5 OR S6
S8	TI (student* or graduat*) OR AB (student* or graduat*)
S9	S7 AND S8
S10	(MH "Rural Health"+) OR (MH "Hospitals and exp Rural"+) OR (MH "Rural Population"+) OR (MH "Rural Health Services"+)
S11	TI (rural* or remote* or region*) OR AB (rural* or remote* or region*)
S12	S10 OR S11
S13	(MH Australia+)
S14	TI (Australia* or Tasmania* or Victoria* or New South Wales or Queensland or Northern Territor* or Western Australia* or South Australia*) OR AB (Australia* or Tasmania* or Victoria* or New South Wales or Queensland or Northern Territor* or Western Australia* or South Australia*)
S15	S13 OR S14
S16	S9 AND S12 AND S15
S17	Limit 16 to yr="2010-Current"

Database(s): APA PsycInfo (EBSCOhost).

Search strategy:.

Query	Search terms
S1	DE nursing OR DE students, nursing
S2	TI (nurs* or nurs* students or midwife* or midwife* students) OR AB (nurs* or nurs* students or midwife* or midwife* students)
S3	DE health occupations OR DE allied health personnel OR DE health workforce
S4	DE students, medical OR DE Physicians OR DE Dentists
S5	TI (medic* or doctor or medical students or doctor students or dentist*) OR AB (medic* or doctor or medical students or doctor students or dentist*)
S6	TI (physiotherap* or speech path* or dietitian* or occupational therap* or podiatr* or social work* or psych* or pharm* or chiropractor* or audiolog* or allied health students or exercise physio* or medical imag*) OR AB (physiotherap* or speech path* or dietitian* or occupational therap* or podiatr* or social work* or psych* or pharm* or chiropractor* or audiolog* or allied health students or exercise physio* or medical imag)
S7	S1 OR S2 OR S3 OR S4 OR S5 OR S6
S8	TI (student* or graduat*) OR AB (student* or graduat*)
S9	S7 AND S8
S10	DE rural health OR DE rural OR DE rural population OR DE rural health services
S11	TI (rural* or remote* or region*) OR AB (rural* or remote* or region*)
S12	S10 OR S11
S13	DE Australia
S14	TI (Australia* or Tasmania* or Victoria* or New South Wales or Queensland or Northern Territor* or Western Australia* or South Australia*) OR AB (Australia* or Tasmania* or Victoria* or New South Wales or Queensland or Northern Territor* or Western Australia* or South Australia*)
S15	S13 OR S14
S16	S9 AND S12 AND S15
S17	Limit 16 to yr="2010-Current"

Database(s): Embase (Elsevier)

Search strategy:.

Query	Search terms
1	Nursing/exp OR "Nursing student"/exp
2	(nurs* OR "nurs* students" OR midwife* OR "midwife* students"):ti,ab,kw
3	'health workforce'/exp OR 'health student'/exp OR 'allied health personnel'/exp
4	'medical profession'/exp OR 'physician'/exp OR 'dentist'/exp
5	(medic* OR doctor* OR "medic* students" OR dentist*):ti,ab,kw
6	(physiotherapy* OR "speech path*" OR dietitian* OR "occupational therap*" OR podiatr* OR "social work*" OR psych* OR pharm* OR chiropractor* OR "allied health students" OR "exercise physio*" or "medical imag*"):ti,ab,kw
7	#1 OR #2 OR #3 OR #4 OR #5 OR #6
8	(student* OR graduat*):ti,ab,kw
9	#7 AND #8
10	"Rural Health"/exp OR "Rural Hospital"/exp OR "Rural Population"/exp OR "Rural Health Care"/exp
11	(rural* OR remote* OR region*):ti,ab,kw
12	#10 OR #11
13	Australia/exp
14	(Australia* OR Tasmania* OR Victoria* OR "New South Wales" OR Queensland OR "Northern Territor*" OR "Western Australia*" OR "South Australia*"):ti,ab,kw
15	#13 OR #14
16	#9 AND #12 AND #15
17	Limit 16 to yr="2010-Current"
