# Supplementary material for: Approaches Used to Describe, Measure, and Analyze Place of Practice in Dentistry, Medical, Nursing, and Allied Health Rural Graduate Workforce Research in Australia: A Systematic Scoping Review
Source: Int J Environ Res Public Health. 2022 Jan 27;19(3):1438. doi: 10.3390/ijerph19031438 (PMC8834932; doi:10.3390/ijerph19031438)
Supplement: Supplementary file 1 [file ijerph-19-01438-s001.zip › Supplementary material3.pdf]

Supplementary Material S3. Excluded studies

Citation	Reason for exclusion
Brown LJ, Macdonald-Wicks L, Squires K, Crowley E & Harris D. An innovative dietetic student placement model in rural New South Wales, Australia	A commentary
O'Sullivan B, Russell DJ, McGrail MR & Scott A. Reviewing reliance on overseas-trained doctors in rural Australia and planning for self-sufficiency: applying 10 years' MABEL evidence. Human Resources for Health. 2019;17(8):1-9.	A review of research
Seal A, Harding C & McGirr J. What influences trainee decisions to practise in rural and regional Australia? Australian Journal of Primary Health. 2020;26(6):520-525.	Combined rural practice intention and place of practice in analysis
Young L, Kent L & Walters L. The John Flynn Placement Program: Evidence for repeated rural exposure for medical students. The Australian Journal of Rural Health. 2011;19(3):147-153.	Combined rural practice intention and place of practice in analysis
Brown L, Crowley E, Harris D & Squires K. Sustaining the rural dietetic workforce in Australia: Outcomes from an immersive rural placement program. Revista Espanola de Nutricion Humana y Dietetica 2016;20:664-665.	Conference abstract
Leung J. Employment for radiation oncologists in Australia and New Zealand - the recent graduates study on employment, experiences, and perspectives. International Journal of Radiation Oncology Biology Physics. 2017;99(2):E126	Conference abstract
Health workers more likely to take rural jobs if they study there or are of rural origin. Australian Nursing & Midwifery Journal. 2021;27(4):5-5.	News article - not peer reviewed
Pearse F & Adamson L. Is there an occupational therapy employment crisis within Australia? An investigation into two consecutive cohorts of occupational therapy graduates from a single Victorian university identifying trends in employment. Australian Occupational Therapy Journal. 2017;65(6):445-476.	No rural focus
Conomos AM, Griffen B, Baunin, N. Attracting psychologists to practise in rural Australia: The role of work values and perceptions of the rural work environment. The Australian Journal of Rural Health. 2013;21(2):105-111.	Not about graduates or year(s) of graduates not reported
Kitchener S. Local regional workforce returns on investment of a locally governed and delivered general practice vocational training program. Australian Health Review: 2020;44(2):254-257.	Not about graduates or year(s) of graduates not reported
Leung J & Kariyasawam S. Employment for radiation oncologists in Australa and New Zealand - Recent graduates study on employment, experiences and perspectives. Journal of Medical Imaging and Radiation Oncology. 2018:62:94-101.	Not about graduates or year(s) of graduates not reported
McGrail MR, Humphreys JS & Joyce CM. Nature of association between rural background and practice location: a comparison of general practitioners and specialists. BMC Health Services Research. 2011;11(63):1-8.	Not about graduates or year(s) of graduates not reported
McGrail MR & Humphreys JS. Geographical mobility of general practitioners in rural Australia. The Medical Journal of Australia. 2015;203(2):92-96.	Not about graduates or year(s) of graduates not reported
McGrail MR, Russell DJ & Campbell DG. Vocational training of general practitioners in rural locations is critical for the Australian rural medical workforce. The Medical Journal of Australia. 2016;205(5):216-21.	Not about graduates or year(s) of graduates not reported
McGrail MR, O'Sullivan BG & Russell DJ. Rural work and specialty choices of international students graduating from Australian medical schools: Implications for	Not about graduates or year(s) of graduates not reported

policy. International Journal of Environmental research and public health. 2019;16(24):1-10.	
Robinson M & Slaney GM. Choice or chance! The influence of decentralised training on GP retention in the Bogong region of Victoria and New South Wales. Rural and Remote Health. 2013;13(2):1-12.	Not about graduates or year(s) of graduates not reported
Taylor SM, Lindsay D & Glass BD. Rural pharmacy workforce: Influence of curriculum and clinical placement on pharmacists' choice of rural practice. The Australian Journal of Rural Health. 2019;27(2):132-138.	Not about graduates or year(s) of graduates not reported
Wearne S, Giddings P, McLaren J & Gargan C. Where are they now? The career paths of the Remote Vocational Training Scheme registrars. Australian Family Physician. 2010;39(1-2):53-56.	Not about graduates or year(s) of graduates not reported
Brown L, Williams L & Capra S. Going rural but not staying long: Recruitment and retention issues for the the rural dietetic workforce in Australia. Nutrition & Dietetics. 2010;67(4):294-302.	Place of practice not an outcome measure
Eley DS, Laurence C, Cloninger CR & Walters L. Who attracts whom to rural general practice? Variation in temperament and character profiles of GP registrars across different vocational training pathways. Rural and Remote Health. 2015;15(3426):1-15.	Place of practice not an outcome measure
Hawthorne L & Hamilton J. International medical students and migration: the missing dimension in Australian workforce planning? The Medical Journal of Australia. 2010;195(5):262-265.	Place of practice not an outcome measure
Keane S, Smith T, Lincoln M & Fisher K. Survey of the rural allied health workforce in New South Wales to inform recruitment and retention. The Australian Journal of Rural Health. 2011;19(1):38-44.	Place of practice not an outcome measure
McGrail MR, Humphreys JS, Joyce CM & Scott A. International medical graduates mandated to practise in rural Australia are highly unsatisfied: Results from a national survey of doctors. Health Policy. 2012;108(2-3):133-139.	Place of practice not an outcome measure
Smith T, Fisher K, Keane S, Lincoln M. Comparison of the results of two rural allied health workforce surveys in the Hunter New England region of New South Wales: 2005 versus 2008. The Australian Journal of Rural Health. 2011;19(3):154-159.	Place of practice not an outcome measure
Sureshkumar P, Roberts C, Clark T, Jones M, Hale R & Grant M. Factors related to doctors' choice of rural pathway in general practice specialty training. The Australian Journal of Rural Health. 2017;25(3):148-154.	Place of practice not an outcome measure
Whiteing N, Barr J & Rossi DM. The practice of rural and remote nurses in Australia: A case study. Journal of Clinical Nursing. 2021;00:1-17.	Place of practice not an outcome measure
Woolley T, Hays R, Barnwell S, Gupta TS & McCloskey T. A successful longitudinal graduate tracking system for monitoring Australian medical school graduate outcomes. Rural & Remote Health. 2015;15(4):1-6.	Place of practice not an outcome measure
Woolley T, Larkins S & Sen Gupta T. Career choices of the first seven cohorts of JCU MBBS graduates: producing generalists for regional, rural and remote northern Australia. Rural and Remote Health. 2019;19(2):1-10.	Place of practice not an outcome measure
Woolley T & Ray RA. Effectiveness of regional medical schools in attracting and retaining students for early-career practice in the local area: The James Cook University experience. The Australian Journal of Rural Health. 2019;27(2):125-131.	Place of practice not an outcome measure
Bailey BE, Wharton RG & Holman CD. Glass half full: Survival analysis of new rural doctor retention in Western Australia. The Australian Journal of Rural Health. 2016;24(4):258-264.	Place of practice not an outcome measure
Catzikiris N, Tapley A, Morgan S, Holliday EG, Ball J, Henderson K, Elliot T, Spike N, Regan C & Magin P. Maintaining capacity for in-practice teaching and supervision of students and general practice trainees: a cross-sectional study of early career general practitioners. Australian Health Review. 2018;42(6):643-649.	Place of practice not an outcome measure

Eaton S, Bonello R, Brown BT & Graham PL. Chiropractice Practice in NSW: A Description of Demographic and Practitioner Characteristics. Chiropractice Journal of Australia. 2012;42(3):114-121.	Place of practice not an outcome measure
Mulcahy AJ, Jones S, Strauss G & Cooper I. The impact of recent physiotherapy graduates in the workforce: a study of Curtin University entry-level physiotherapists 2000-2004. Australian Health Review. 2010;34(2):252-259.	Place of practice not an outcome measure
Russell DJ, Humphreys JS, McGrail MR, Cameron WI & Williams PJ. The value of survival analyses for evidence-based rural medical workforce planning. Human Resources for Health. 2013;11(65):1-9.	Place of practice not an outcome measure
Buttner R, Blakely N, Curtin S, Wall B, Pougnault S, Burkitt T & Playford D. What does 'rural return' mean? Rural-origin medical graduates do not 'go home'. Rural and Remote Health. 2017;17(2):1-3.	Research letter
Gerber JP & Landau LI. Driving change in rural workforce planning: the medical schools outcomes databases. Australian Journal of Primary Health. 2010;16(1):36-39.	Rural practice intention as outcome measure
Jones M, Humphreys JS & McGrail MR. Why does a rural background make medical students more likely to intent to work in rural areas and how consistent is the effect? A study of the rural background effect. The Australian Journal of Rural Health. 2012;20(1):29-34.	Rural practice intention as outcome measure
Kirschbaum M, Khalil H, Talyor S & Page AT. Pharmacy students' rural career intentions: Perspectives on rural background and placements. Currents in Pharmacy Teaching and Learning. 2016;8(5):615-621.	Rural practice intention as outcome measure
Kaur B, Carberry A, Hogan N, Roberton D & Beilby J. The medical schools outcomes database project: Australian medical student characteristics. BMC Medical Education. 2014;14:180.	Rural practice intention as outcome measure
Johnson G, Wright FC, Foster K & Blinkhorn A. Rural placement experiences in dental education and the impact on professional intentions and employment outcomes- a systematic review. European Journal of Dental Education. 2018;22(3):364-378.	Sub-studies already included in search
Johnson GE, Wright FC & Foster K. The impact of rural outreach programs on medical students' future rural intentions and working locations: a systematic review. BMC Medical Education. 2018;18(1):1-19	Sub-studies already included in search
