# Supplementary material for: Approaches Used to Describe, Measure, and Analyze Place of Practice in Dentistry, Medical, Nursing, and Allied Health Rural Graduate Workforce Research in Australia: A Systematic Scoping Review
Source: Int J Environ Res Public Health. 2022 Jan 27;19(3):1438. doi: 10.3390/ijerph19031438 (PMC8834932; doi:10.3390/ijerph19031438)
Supplement: Supplementary file 1 [file ijerph-19-01438-s001.zip › Table S1.pdf]

Table S1. Characteristics of included studies.

Citation	Discipline	Scale [state/ territory/ national)	Study design (data collection methods)	Study aim(s)	Outcome measure(s)	Approach to measuring, describing or analyzing place of practice	Participant demographics (n, %F, age)/years of graduation	Response rate/ proportion of cohort included	Results	Implications of findings
**Rural, Remote and Metropolitan Areas Classification (RRMA)**										
Johnson, Wright & Foster 2019[34]	Dentistry	University specific (NSW)	Cross-sectional study (survey, AHPRA, google)	To evaluate the impact of a Rural Clinical Placement Program (RCPP) offered to final year dental students, on the graduates working locations.	Work locations	The RRMA classification system was used with metropolitan employment categorized as RRMA1-2 and rural employment (RRMA3-5).	n=135; 47% F; 70% 26-30 years (2009-2013)	33%	One third of RCPP graduates (33.3%) were working in a rural location, compared to 17.8% of non-RCPP graduates. The odds of working in a rural setting in 2015, were higher for RCPP graduates when compared to those who did not participate in the program (OR=1.83, 95% CI 1.00, 3.56).	Graduates who participated in the RCPP were more likely to work in rural Australia in 2015 to 2016.
Clark et al. 2013[36]	Medicine	University specific (NSW)	Longitudinal study – three cohorts (repeated survey)	To determine whether recruitment of rural students and uptake of extended rural placements are associated with students' expressed intentions to undertake rural internships and students'	Intention to undertake a rural internship and acceptance of rural internship	Locations were mapped to RRMA categories and grouped as urban (M1, M2) or rural (R2-R3).	n=448; not reported; not reported (commenced 2005-2007)	55%	The proportion of students preferring a rural career decreased from 20.7% to 12.5% between entry and exit. 8.1% accepted a rural internship. Students who had undertaken an extended rural placement were more than twice as likely to accept a	Rural clinical training through extended placements had a stronger association when compared to a rural background with a preference for, and acceptance of, a rural internship.

				acceptance of rural internships after finishing medical school, and to compare any associations.					rural internship when compared to those with a rural background.	
Johnson & Blinkhorn 2013[35]	Dentistry	University specific (NSW)	Longitudinal cohort (AHPRA)	To monitor the location of recent graduates who undertook a rural clinical placement whilst in the final year of Sydney Dental School, and to determine whether it influenced their choice of job location.	Work locations	Rural, remote and metropolitan areas (RRMA) classification system was used to classify which areas of Australia were metropolitan, regional, rural and remote zones. RRMA 1-2 were classified as metropolitan and RRMA 3-5 as rural.	n=158; not reported; not reported (2008-2009)	Not reported.	12.7% of 2008 graduates were employed in rural location compared to 27.6% of 2009 graduates. In 2011, of the 2009 graduates who participated in the rural placement, 44.8% were working in rural or remote region compared to 17% of graduates who did not participate.	Graduates who participated in a rural placement program were more likely to be working rurally when compared to other graduates.
Kitchener et al. 2015[37]	Medicine	University specific (QLD)	Retrospective cohort study (University data and Queensland Health data)	To assess outcomes in terms of academic performance and subsequent career choice in medical students undertaking a longitudinal integrated rural clinical placement.	Academic and clinical performance, intern location	RRMA levels 3-7 were classified as rural.	n=472; not reported; not reported (2010-2014)	67%	When performance in prior year was considered, there were no statistically significant differences in academic performance in year 3 or 4 for rural and urban students. Of graduates who had undertaken a longitudinal rural placement, 67%	Implementing a longitudinal integrated rural clinical placement may increase the likelihood of graduates practicing in rural settings.

								completed an internship at a rural location compared with 15% for urban hospital-based students (odds ratio 11.91; 95% confidence interval 6.08–23.32).	
Playford & Cheong 2012[38]	Medicine	University specific (WA)	Retrospective longitudinal cohort study (University of Western Australia data)	To examine the working locations of PGY1 and PGY2 practitioners and, through linkage, compares this with their undergraduate experience of rural practice and history of rural life.	Practice location postgraduate.	A placement was included if four weeks or longer and classified as rural if in RRMA 3-5. No placements were undertaken in RRMA 6 and RRMA 7.	n=490; not reported; not reported (enrolled between 1997-2001)	72%	Participation in the RCS program was associated with significantly more postgraduate year one rural work than shorter rural placements alone (OR = 1.5, CI 0.97–2.38).	Rural initiatives, in particular the RCS program, are associated with postgraduate rural choices.
Strasser et al. 2010[39]	Medicine	University specific (VIC)	Retrospective cohort study (survey)	To determine if selecting rural background students into the Monash MBBS program affects vocational training location and intended practice location after training.	Intent towards rural practice, vocational training location and practice location.	Postcodes were classified using the seven RRMA categories. Dichotomized into RRMA1 and 2, and RRMA3-7.	Rural background: n=83; urban background: n=140; 62% F; 28 (mean) (students 1992-1999)	Rural: 52%; urban 36%	There was a positive, but non-significant association between rural background and RRMA 3–7 community as their current location and first place of practice once vocationally qualified.	Interest in rural practice is not fully reflected in location during or after vocational training. The beneficial effects of rural undergraduate exposure might be lost if internship and vocational training programs provide insufficient rural clinical

									experiences and curriculum content.
**Australian Standard Geographical Classification Remoteness Areas (ASGC-RA)**									
Gupta et al. 2019[42]	Medicine	University specific (WA)	Longitudinal study (repeated survey)	To determine the number and duration of tours of service for Rural Clinical School graduates and by post-graduate year.	Number of rural service tours (period of at least two weeks duration and at least one year spent working in a rural area within a calendar year) and duration	The ASGC-Remoteness Areas were used, with rural defined as RA2-5.	n=468; 64% F; 71% <25 years (2002-2011)	96%	A total of 17,786 weeks were spent working rurally for at least two weeks by 239 graduates from 2006 to 2015. 120 graduates completed at least one rural tour, with a mean length of 1.89 years (1.69 - 2.10 years).	Rural Clinical School graduates were found to undertake a significant amount of rural work in years following graduation (specifically PGY 3-12), however relatively low rates of sustained rural practice were identified.
Herd et al. 2017[43]	Medicine	National (AU)	Longitudinal study (Medical Schools Outcomes Databases (MSOD) longitudinal tracking database)	To identify factors mediating with the preferences and rural work of Australian medical graduates.	Preferred and actual work locations of graduates one (PGY1) and three years (PGY3) post-graduation.	The ASGC-Remoteness Areas were used, with rural defined as RA2-5.	n=4028; 56% F; 28 (mean) (2006-2013)	19%	At PGY1 and PGY3, 19% and 22% of respondents respectively, reported a preference for rural practice, whereas 41% and 51% reported completing a rotation in a rural location.	Rural preference is determined prior to medical school and was identified as the most consistent predictor of completing a rural rotation in years one and three, post-graduation.
Kitchener 2021[44]	Medicine	Local health district (QLD)	Longitudinal study (self-reported data, AHPRA)	To examine and evaluate the retention of medical graduates for a hospital and health service partnering with a university to	Work locations.	The ASGC-RA was used to classify work locations as regional (ASGC-RA2-5) or metropolitan (ASGC-RA1).	n=119; not reported; not reported (2010-2017)	98%	Regional internships were undertaken by 60% of graduates, with 31% of graduates working with the local health service with rotations to rural generalist	Investment in clinical training for rural generalists in local health districts can produce retention of medical graduates in local areas.

				deliver a rural medical program.					hospitals. Students who had spent two years on the program with the local health service were more likely to remain local for an internship (OR 5.7) and be practicing local after the internship (OR 3.3).	
Kwan et al. 2017[45]	Medicine	University specific (QLD)	Cross-sectional study (survey)	To determine the predictors of longer-term rural practice, for GPs and specialists.	Primary place of graduates' practice.	Rural practice was categorized as ASGC-RA2-5, with longer-term rural practice categorized as 50% of time since graduation in a rural location.	n=744; 52% F; 33.3 years (2002-2011)	48%	Predictors of long-term rural practice were a rural background (OR 2.10, 1.37-3.2) and a bonded rural scholarship (OR 2.11, 1.19-3.76).	A combination of rural background and rural undergraduate clinical training increases the probability of specialislts and general practitioners in undertaking rural practice.
Mc Grail et al. 2017[11]	Medicine	National (AU)	Longitudinal study (MABEL survey)	To explore the association between career stage and rural medical workforce supply among Australian medical graduates.	Proportion of general practitioners and specialists working in rural locations according to career stage, gender and origin (rural versus metro-politan).	Rural origin was classified as ASGC-RA 2-5 for at least 6 years, whereas work location was classified as metropolitan (RA1) or rural (RA2-5).	n=9187; 45% F; not reported (1970 onwards)	Not reported.	Establishing and early career GPs had a significantly greater likelihood of working rurally (OR 1.67 and 1.38, respectively). Women were likely to be rural GPs, but less likely to be rural specialists. Rural-origin was associated with higher odds of rural practice.	The supply of the rural medical workforce continues to be challenging despite significant investment.

Moore et al. 2018[46]	Medicine	University specific (ACT)	Cross-sectional (AHPRA)	To determine the location of RCS graduates from the Australian National University Medical School.	Work location data for graduates.	Rurality was classified using the ASGC-RA; RA1 for metropolitan centres and RA2-5 for increasing rurality and remoteness.	n=965; 59% F; not reported (2007-2017)	95%	A higher proportion of graduates after their fifth postgraduate year worked in a rural location when compared to graduates in the first five years of practice (34.7% compared to 15.2%, respectively).	Graduates in the first five years spend time in metropolitan centres before returning to work in rural areas.
Playford & Puddey 2017[47]	Medicine	University specific (WA)	Cohort study (AHPRA, University of Western Australia medical school data)	To compare the employment location of medical graduates who had no rural interest as undergraduates with those who applied but did not participate in the rural clinical school, and those who applied and participated in the rural clinical school.	Graduates' current principal workplace location.	ASGC-RA; RA2-5 rural, RA1 urban	Did not apply for RCS n=593; 50.6% F; 58.7%; ≥24 years: applied for but did not enter RCS n=107; 56.1% F; 72.9% ≥24 years: applied for and entered RCS n=275;64.4% F; ≥24 years 60.4% (2004-2011)	Not reported.	Compared to those who did not apply for RCS, those who applied for or entered the RCS were two fold more likely to work in a rural location (OR 2.02, 95% CI 1.04, 3.93) whilst those completing the RCS were nearly fourfold more likely to be working rurally (OR 3.82, 95% CI 2.46, 5.93).	RCS graduates are more likely to work rurally compared to other student cohorts, suggesting the RCS has an independent effect on rural workforce.
Playford et al. 2017[48]	Medicine	University specific (WA)	Cohort study (Medical School Outcomes Database Commencing Medical Students Questionnaire (CMSQ),	To compare the influence of rural background, rural intent at medical school entry and RCS participation on	Medical practice in rural areas during postgraduate years 2-5.	ASGC-RA; RA2-5 rural, RA1 urban	n=569; RCS 70% F: urban 56% F; not reported (commenced medical school 2006-2010)	64%	Students of rural origin were four times as likely as urban origin students to practice rurally following graduation (OR 4.3, 95% CI 2.4-7.8).	Rural background, rural intention and RCS participation should all be considered by medical schools seeking to address the maldistribution

			University of Western Australia data and AHPRA)	later rural practice					RCS participants were more than twice as likely to work rurally, compared with other students (OR 2.3, 95% CI, 1.3-4.1).	of the medical workforce in rural areas.
Playford et al. 2019[49]	Medicine	University specific (WA)	Cohort study (University of Western Australia data, AHPRA)	To compare rural workforce outcomes for three separate streams of medical students: school leaver entry, non-standard entry with some prior tertiary education and graduate entry.	Rural practice.	ASGC-RA; RA2-5 rural, RA1 urban	n=1122; school leaver 45% F: 23.6; non-standard entry 39.8% F: 26.9; graduate entry 39.7% F: 29.9 (2004-2015)	96%	Both non-standard entry and graduate entry medical students had an increase in the odds of practicing rurally when compared to school leaving entry students (OR 3.89, 95% CI 2.29, 6.60 and OR 2.22, 95% CI 1.42, 3.46, respectively).	Graduates with prior tertiary education were more likely to practice rurally when compared with school entrants.
Playford, Burkitt & Atkinson 2019[50]	Medicine	University specific (WA)	Cohort study (University of Western Australia data, AHPRA)	To examine the workforce characteristics of graduates from the Rural Clinical School of Western Australia using Social Network Analysis.	Primary practice location and RCS site of origin.	ASGC-RA; RA2-5 rural, RA1 urban (excluded from study)	n=709 (PGY2-PGY15); not reported; not reported (2001-2014)	Not reported.	Of graduates included, 17.5% were in rural practice in 2017, with nearly two thirds of these located in rural WA. The location of rural practicing graduates was largely in the north of Australia.	The RSCWA was considered a weak tie in SNA, with graduates working in rural areas they had little long-term prior contact with - generally in the north of the state.
Playford, Moran & Thompson 2020[70]	Nursing and allied health	University specific (WA)	Longitudinal cohort study (AHPRA, online survey, Western	To measure long term (15-17 years) rural practice outcomes for	Practice location in 2018.	ASGC-RA: major cities RA1, other locations classed as rural.	n=244; 67% F; age not reported (2000-2003)	31%	Of graduates included 15-17 years following their rural	Initial rural practice is a significant predictor of working rurally

			Australia UDRH data)	nursing and allied health graduates who had completed an undergraduate rural placement of 2-18 weeks through a UDRH.					placement, most were practicing in RA1, with the remainder in RA2. RA3 and RA 4-5, with 20% practicing rurally. A statistically significant association was identified between the region practicing in the year after graduation and the region practicing 15-17 years following graduation.	long term for nursing and allied health graduates. Supporting careers in rural nursing and allied health rural training is important to redress the maldistribution of the health workforce in rural and remote areas.
Playford, Ngo & Puddey 2021[51]	Medicine	University specific (WA)	Longitudinal cohort study (MSOD survey, AHPRA)	To examine rural and urban workplace intention changes and actual rural versus urban work.	Rural intentions and practice location.	ASGC-RA: major cities RA1, other locations classed as rural.	n=547; 60% F; 53% <25 years at graduation (2006-2016)	51%	Of included graduates, 17% practiced rurally. Rural practice intention and rural background/RCSWA, were significant statistical predicators of actual rural practice (p<0.0001).	Rural intention and RCSWA experience were predictors of rural practice.
Playford, Ngo, Atkinson & Puddey 2019[52]	Medicine	University specific (WA)	Longitudinal cohort study (University of Western Australia data, AHPRA)	To examine the practice location of graduates to investigate longitudinal trends in rural and remote practice and	Annual proportion of graduates practicing rurally, annual proportions of graduates practicing more	ASGC-RA: major cities RA1, other locations classed as rural.	n=878; 55% F; 69.8% <25 years at graduation (2004-2010)	87%	Rural origin and RCS graduates had the highest proportions in rural areas at all timepoints from 2013 to 2018 (OR 9.70 95% CI 5.41,	RCS graduates and rural origin students contribute to the rural and remote health workforce.

				compared these among graduates with different backgrounds (rural v. urban; RCS v. non-RCS).	remotely (RA3-5) and mean cumulative duration in years in rural practice				17.40) when compared to the urban reference group. These graduates also had a cumulative duration of rural practice five fold that of the urban reference group.	
Woolley, Gupta & Bellei 2017[53]	Medicine	University specific (QLD)	Cross-sectional study (James Cook University (JCU) tracking database of graduates)	To describe factors predicting JCU medical graduates undertaking at least one year of remote practice.	Post graduate location and specialist training.	The ASGC-RA was used to categorize locations as either non-remote (RA1-3) or remote (RA4-5).	n=529; 60% F; 23 (median age at graduation) (2005-2011)	99%	9% of the JCU medical graduates had practiced for at least one year in a remote location between PGY 4 and 10. Remote practice was predicted by completing rural generalist training (p<0.0001, prevalence odds ratio 17.0), being awarded an 'above average' interview score at medical school selection (p=0.006, POR 5.1), attending the Darwin clinical school in years 5-6 (p=0.005, POR 4.7), being female (p=0.016, POR 3.6), and undertaking outer-regional or remote internship (p=0.006, POR 3.5).	Investment in medical education through clinical skills and internships in rural or remote locations, may be potential strategies to increase the remote medical workforce.

Woolley, Gupta & Larkins 2018[54]	Medicine	University specific (QLD)	Cross-sectional study (online survey and MABEL study)	To compare the range of work settings and hours worked by James Cook University medical graduates to Australian medical graduates.	Work settings and hours worked, including rural practice.	Not described in methods but presented place of practice presented in results using ASGC-RA collapsed into RA1 'major cities', RA 2 'Inner regional Australia' and RA3-5 'outer regional Australia', 'remote Australia', and 'very remote Australia'.	n=180; 64% F; 92% <35 years (2005-2011)	34%	7% of graduates were working in ACCHOs located in RA2-5. 43% of postgraduates were working in RA1, 21% in RA2 and 36% in RA3-5.	Graduates were working mostly in public settings, including primary care settings and ACCHOs which was identified as appropriate for the predominately rural and remote geography of Queensland.
Brown et al. 2017[69]	Allied Health	University specific (NSW)	Mixed methods cohort study (qualitative interviews, end of placement survey and Rural Allied Health Workforce Survey)	To evaluate allied health students' experiences of their short-term, medium-term, or long-term rural placement and to follow their career outcomes.	Undergraduate placement experience and other measures including postcode of their current place of employment.	The ASGC-RA index was used to classify all postcodes as: major cities (RA1), inner regional (RA2), outer regional (RA3), remote (RA4) and very remote (RA5).	n=80 (follow up survey); 75% F; 47.5% <24 years (2011-onwards)	40%	Graduates (n=80) followed up one year after graduation were mostly employed in rural or remote locations (50%). The largest number who were employed in rural areas were dietitians. Graduates who described their background as rural were 2.63 times (95% CI 0.96-7.28) were more likely to be	Those with a rural background were more likely to practice rurally.

									employed in a rural or remote area (RA2-5) than those who described their background as metropolitan.	
Lewis et al. 2016[55]	Medicine	State-wide (NSW)	Cross-sectional (online survey)	To report on the results of an evaluation undertaken in 2014 of the New South Wales Rural Resident Medical Officer Cadetship Program.	Place of practice 2014.	Geographical information (where cadets grew up and location of practice in 2014) was categorized using the ASGC-RA. In this study, categories RA2-5 are described as rural.	n=142; 59% F; 25 (mean) (entered program 1989-2010)	67%	Over half of the cadets included in the analysis (n=74, 53%) were working in rural areas (ASGCRA 2-5) in 2014 and practice location was significantly ($p<0.001$) influenced by career choice. Of the cadets working in rural locations, the majority (58%) were working as general practitioners while 38% had chosen other specialties and 4% were working as hospital non-specialists.	Findings supported the cadetship being a link for medical graduates to rural practice.
Playford, Ng & Burkitt 2016[56]	Medicine	University specific (WA)	Longitudinal cohort study (survey)	To follow the workforce choices of 10-years of graduates from a longitudinal rural immersion Programme.	Rural work location	Use of ASGC-RA but no information as to how rural was classified.	n=417; not reported; not reported (2003-2012)	89%	16-50% of contacted alumni worked rurally for a period of each post-graduate year. Aggregated over time, the majority took up to 30% of	Undergraduate rural immersion is sufficient to create a graduate rural workforce that is mobile.

									their postgraduate training rurally.	
Eley et al. 2012[57]	Medicine	University specific (QLD)	Longitudinal mixed methods study (survey)	To monitor the impact of rural undergraduate clinical training on trends in workforce participation patterns of its graduates as long as 9 years in the workforce.	Workforce participation including location.	Using the ASGC-RA classification, location was classified as urban (RA1) or non-urban (RA2-5).	n=115; 50% F; 30-34 88% (2002-2009)	64%	Of respondents, 40% were working in non-urban locations.	The drivers influencing the decisions of medical graduates to practice rurally are personal/family reasons, a positive rural exposure and specialty training requirements.
Hogenbirk et al. 2015[58]	Medicine	University specific (VIC)	Cohort study (repeated survey)	To test predictors of practice location of fully qualified Monash University Bachelor of Medicine, Bachelor of Surgery (MBBS) graduates.	First practice location, current practice location and intended practice location.	Due to small sample sizes, ASGC-RA1 (major city) was compared with ASGC-RA2-5 (rural and remote).	n=153; 60% F; 33.8 (mean) (1992-1999)	Rural 52%; urban 45%	Having a rural background compared to an urban background was a significant predictor of rural first practice location and rural current practice location.	The rural background effect reduced with time, highlighting the need for continued support during postgraduate training.
Jamar, Newbury & Mills 2014[59]	Medicine	University specific (SA)	Retrospective cohort study (online survey)	To track the early career movements of these graduates in order to determine the program's rural medical workforce impact.	Work locations.	The ASCG-RA was used. Rural categorized as RA 2-5.	n=74; not reported; not reported (2004-2011)	58%	Between 2009 and 2012, 20.8% to 34.1% of respondents were located in a rural area (ASGC - Remoteness Areas 2-5). More than half of respondents spent time in a rural area since graduation.	Rural clinical training programs can supplement an initial interest in rural practice.

Kondalsamy-Chennakesavan et al. 2015[60]	Medicine	University specific (QLD)	Retrospective cohort study (survey, AHPRA)	To determine the role of rural background and years of rural clinical school training on subsequent rural clinical practice.	Current clinical practice location.	The category ASGCRA1 was considered metropolitan, and categories ASGC-RA2 to ASGCRA5 were considered rural (RA2, inner regional; RA3, outer regional; RA4, remote; RA5, very remote). If location of current clinical practice was overseas, it was categorized as metropolitan	n=754; not reported; not reported (2002-2011)	48%	Clinical practice location was rural for 18.8% of metropolitan clinical school attendees and 41.7% rural clinical school attendees (P < 0.001).	Rural background and 1-2 years of rural training are independent predictors of rural practice.
Playford et al. 2014[61]	Medicine	University specific (WA)	Cohort study (AHPRA)	To determine whether completing a year of the RCSWA program is associated with entering the rural medical workforce.	Rural or urban work location of graduates.	ASGC-RA - Graduates were designated as working rurally if their primary practice location was in an area defined by the ASGC-RA as 2–5, and city/urban if ASGC-RA 1.	n=1017; 53% F; 29.7 (median) (graduated by 2010)	91%	Of 258 RCSWA graduates, 16.3% were working rurally compared with 4.7% of controls. Of 195 RCSWA graduates from urban backgrounds, 14.9% were working rurally compared with 3.8% of urban background controls. Of 63 rural-background	Participation in the RCSWA is strongly associated with a greater likelihood of working rurally.

									RCSWA graduates, 20.6% were working rurally, compared with 14.7% of rural-background controls. Using logistic regression, RCSWA participation had a strong relationship with working rurally.	
Playford et al. 2015[7]	Medicine	University specific (WA)	Cohort study (AHPRA)	To explore the hypothesis that amongst UWA graduates who are currently working in rural practice, those who completed a longitudinal rural clerkship in the RCSWA are more likely to be practicing in more remote locations than graduates who completed all their training in urban areas.	Likelihood of graduates practicing in more remote, versus inner regional, locations.	Graduates were designated as working rurally if their primary practice location was in an area defined by the ASGC-RA as 2–5, and city/urban if ASGC-RA 1.	Pre-RCSWA: n=200; 41% F; 24 ≤ 53%. RCSWA: n=63; 54% F; 24 ≤ 81%. Post RCSWA: n=61; 67% F; 24 ≤ 66% (1980-2011)	Not reported.	The two groups not participating in the RCSWA had 45.5% and 52.4% of participants in outer regional/very remote locations, respectively. In comparison, 78.7% of those who had participated in the RCSWA were currently practicing in outer regional/very remote locations.	Extended rural clinical clerkship during an undergraduate medicine course is related to a much greater likelihood of practicing in more remote, under-serviced rural locations.
Puddey et al. 2015[62]	Medicine	University specific (WA)	Cohort study (AHPRA, University of Western Australia data)	To assess the extent to which medical school selection criteria and/or the socio-demographic	Practice location.	Graduates were designated as working rurally if their primary practice location was in an area	n=729; 55% F; 23.6 (mean) (graduated by 2011)	94%	Those practicing in a rural location in 2014 were more likely to have come from the lower 6 IRSAD deciles (OR	The major focus in attempts to grow the rural medical workforce should therefore remain on recruiting medical

				profile of medical students may further facilitate or hamper the selection of students ultimately destined for the rural medical workforce.		defined by the ASGC-RA as RA 2–5, and urban if RA 1.			2.75, 95% CI 1.44, 5.23, P = 0.002), to be older (OR 1.86, 95% CI 1.09, 3.18, p = 0.023) and to have a lower UMAT-3 (Non-verbal communication) score (OR 0.98, 95% CI 0.97, 0.99, P = 0.005).	students from a rural background together with providing maximal opportunity for prolonged immersion in rural clinical environments during their training.
Ray, Woolley & Sen Gupta 2015[63]	Medicine	University specific (QLD)	Cohort study (AHPRA, personal contact, University data)	To determine if the JCU medical school's policy of preferentially selecting rural and remote background students is associated with differing patterns of undergraduate performance or graduate practice location.	Graduate practice location.	Graduates who did not spend an entire calendar year in one location were classified according to the self-defined location they provided when asked where they had spent the majority of the year. The term 'rural' is used for RA 3, and the term 'remote' for RA 4 and 5.	n=744; not reported; not reported (2005-2013)	97%	Graduates having either a rural or a remote hometown at application were more likely to practice in rural (RA 3-5) towns than graduates from metropolitan/inner regional centre across all postgraduate years.	These data suggest that JCU's selection process favouring rural origin applicants does not compromise academic standards, but does produce graduates whose patterns of practice accord with the mission of the school.
Schauer, Woolley & Sen Gupta[64]	Medicine	University specific (QLD)	Cross-sectional study (survey)	To identify the main reason JCU MBBS graduates chose their internship location and first four practice relocations.	Graduates' main reason for choosing internship location and up to four subsequent relocations, post-graduate	The practice city/town for both internship year and for 2012 was categorized into 'metropolitan' (RA 1), or 'non-metropolitan'	n=175; not reported; not reported (2005-2009)	67%	Personal factors were the primary driver for choosing their internship location with 33% returning to 'near their family/home town', and 21% staying in the town	Personal decisions take precedence over professional career decisions for why JCU medical graduates chose their internship location, but subsequent

					specialty training undertaken and practice location (either metropolitan or non-metropolitan) for graduates' internship year and current practice year (2012).	(RA 2–5), based on the ASGC-RA index.			they were based in Years 5 and 6. Internship location was not associated with later specialty training, but current metropolitan practice was associated with Surgical or Paediatrics training (P = 0.007 and P = 0.063, respectively), while current non-metropolitan practice was associated with General Practice and Rural Generalist training (P = 0.010 and P = 0.001, respectively)	relocations are driven by career ambitions, usually around specialty training requirements.
Sen Gupta et al. 2013[65]	Medicine	University specific (QLD)	Cohort study (repeated surveys and MSOD survey)	To report on progress towards achieving the School's mission of positively influencing graduates' rural career intentions.	Rural career intention and actual internship location.	Internship locations in both surveys were categorized using the ASGC-RA classification system. Data is presented as capital city, major urban pop>100000, regional city/town pop 25000-100000,	n=292; not reported; not reported (2005-2010)	66%	At graduation, 88% of JCU medical students intended to practice outside capital cities compared to 31% graduates from other medical schools (odds ratio (OR): 16.5). 67% of JCU graduates undertook their internship outside a metropolitan centre compared	Data suggests that career outcomes of JCU medical graduates are aligned with the workforce needs of the region, and different from those graduating from Australia's predominantly metropolitan medical schools.

						smaller town <25000.			with 17% of others (OR:10.0), and 47% in outer regional centres compared with 5% (OR: 16.6), respectively.	
Sen Gupta et al. 2014[66]	Medicine	University specific (QLD)	Longitudinal cohort study (University data, personal data, AHPRA)	To present the early career practice locations and the specialty training undertaken by these first seven cohorts from JCU SoM, and describes the association between later practice location with both hometown at application and internship location.	Practice location.	did not spend an entire calendar year in one location were classified according to the self-defined location they provided when asked where they had spent the majority of the year. The term 'nonmetropolitan' was used for categories RA 2–5, and the term 'rural' for categories RA 3–5.	n=536; not reported; not reported (2005-2011)	99%	A total of 65% of JCU graduates undertook their internship in non-metropolitan locations including 20% in RA 2 and 44% in RA 3–5, a pattern of practice different to that of other Australian clinicians.	This early evidence supports the JCU model of distributed non-metropolitan medical education, and suggests more regionally-located internship and specialty training places would further increase the medical workforce in northern and/or rural Australia.
Woolley et al. 2014[67]	Medicine	University specific (QLD)	Longitudinal cohort study (University data, AHPRA)	To describe factors predicting JCU medical graduates having a rural practice location at postgraduate year (PGY) 5.	Predictors of practice in a rural town ASGC-RA 3–5 at PGY 5.	Categorized into 'metropolitan', 'inner regional' and 'outer regional or remote' based on the ASGC-RA index.	n=264; 58% F; 23 (median) (2005-2008)	98%	Practice in a 'rural' town in PGY 5 was predicted by 'internship in an ASGC-RA 3–5 location' (prevalence odds ratios (POR) = 3.9, P < 0.001), 'undertaken postgraduate training as a	Investment in rural medical education will produce an appropriately trained medical workforce to meet the needs of rural Australia. Rural workforce may be further enhanced with additional specialty training

								general practitioner or rural generalist' (POR = 3.4, P < 0.001), 'hometown at application located in ASGC-RA 3–5 area' (POR = 2.9, P = 0.023), 'Aboriginal & Torres Strait Islander ancestry' (POR = 5.6, P = 0.031), and NOT undertaken post-graduate training in surgery (POR = 5.4, P = 0.055).	opportunities in rural tertiary hospitals, in particular, surgery.
**Australian Statistical Geography Standard (ASGS) Remoteness Structure**									
Bacopanos & Edgar 2016[72]	Physiotherapy	University specific (WA)	Cross-sectional study (online survey)	To determine the employment patterns of physiotherapy graduates from the University of Notre Dame.	Employment location, employment status, health care sector, area of practice, and salary and employment history.	Postcodes were assigned to regions as per the Australian Government Department of Health classifications: Major City, Inner Regional, Outer Regional, Remote, Very Remote.	n=157; 71% F; 27.4 years (mean) (2006-2012)	50%	Of n=146 participants who provided their postcodes, most (n=119, 82%) worked in a Major City, with fewer in Inner Regional (n=8), Outer Regional (n=7), Remote (n=1) and Very remote locations (n=4). There were 7 participants working overseas.	A greater uptake of employment in rural areas was found for graduates. However, graduates were not retained in rural areas. A greater focus on the retention of physiotherapy graduates in rural areas is recommended.
Duffy et al. 2021[73]	Optometry	National (AU)	Cohort study (AHPRA principal place of practice data)	To examine the initial work location of new entrants to the	Qualification, registration type and	The ASGS classification was used and locations were	n=1680; 66% F; not reported (2010-2018)	Not reported.	The majority of new entrants (75%) to optometry listed their principal	The majority of graduate optometrists in

				optometry profession.	principal place of practice.	categorized as Major Cities of Australia (RA1), Inner Regional Australia (RA2), Outer Regional Australia (RA3), Remote Australia (RA4), and Very Remote Australia (RA5).			place of practice in a major city (RA1).	Australia practice in Major Cities.
Sutton et al. 2021[74]	Nursing and allied health	University specific multiple (VIC and NSW)	Cohort study (data linkage: AHPRA, Monash University data, Newcastle University data)	To examine nursing and allied health graduate entry into rural practice.	Principal place of practice	Principal place of practice was dichotomized using the ASGS-RA: RA 1 categorized as 'major city', and RA2-5 categorized as 'rural'.	n=1130; 81.1% F; 61.9% age <21 years at enrolment (2017)	Not reported.	There was no difference in the geographical distribution of disciplines across 'Major City' and 'Rural' areas. Between 78% and 83% of graduates had taken up working in a 'Major City'. At the time of their second year registration, 18% worked in a rural principal place of practice. Rural origin graduates were 4.45 times more likely to work in a rural principal place of practice compared to urban origin graduates.	Factors influencing graduate rural principal place of practice include rural origin and more rural placement days.
Fuller, Beattie & Versace 2021[75]	Medicine	University specific (VIC)	Cross-sectional (AHPRA, Deakin University data)	To determine the student characteristics	Graduates' work locations.	Locations classified as major cities of	n=948; 49% F; not reported (2011-2018)	93%	83.8% were working in metropolitan	A combination of rural background and extended rural

				and clinical school training pathways that are associated with postgraduate rural practice		Australia (RA1) were classified as metropolitan and locations classified as inner regional Australia, outer regional Australia, remote Australia and very remote Australia (RA2-RA5) were classified as rural.			Australia (RA1) and 16.2% were working in rural locations (RA2- 5). Rural background students twice as likely to work rurally when other potential confounders (eg rural return of service obligations) were controlled for.	training are key strategies to increase the likelihood of medical graduates practicing rurally.
**Modified Monash Model (MMM)**										
Campbell et al. 2019[76]	Medicine	University specific (VIC)	Longitudinal study (university data and AHPRA principal place of practice data)	To compare the work locations following registration of medical students who had completed one year of a longitudinal integrated clerkship (LIC), with other students (non-LIC, non-LIC rural training).	Graduates' main work location	Work location geocoded using town name and postcode and categorized using MMM with metropolitan as MMM1 and rural as MMM2-7. The categories of 'large regional' (MMM2) and 'smaller regional and rural towns' (MMM3-7) were also used.	LIC: n=130; 55% F; 70% ≤ 19 years at course entry. Non-LIC: n=519; 62% F; 63%≤ 19 years at course entry. Non-LIC rural: n=1763; 52.2% F; 66% ≤ 19 years at course entry. (2008-2016)	99%	Medical students who had undertaken a fourth year LIC in addition to other rural training, were more likely to work in smaller regional or rural towns when compared to other students (RRR 5.62, 95% CI 2.81-11.20).	Findings supported the value of rural LICs in addition to rural training, to address the maldistribution of the medical workforce in rural Australia.
McGrail et al. 2020[77]	Medicine	State-wide (VIC)	Cohort study (Postgraduate Medical Council of Victoria administrative dataset)	To describe and compare preferences for, and uptake of rural internships by graduates	Preferred internship location and accepted location of internship.	Internship locations geocoded using MMM, with MMM1 as metropolitan,	n=4562; not reported; not reported (2012-2016)	Not reported.	Domestic graduates filled the majority of rural internship positions (69.7%) and metropolitan	Regional areas must rely on international graduates as preferences for and uptake of rural

				from Victorian medical schools.		MMM2-5 as rural. Rural locations were stratified as MMM2 'large regional' and MMM3-5 'smaller regional or rural towns'			positions (92.2%). Of graduates, 20.1% included a rural location in their top five preferences.	internship positions by domestic graduates are not sufficient to meet the demands of the rural medical workforce.
McGrail, O'Sullivan & Russell 2018[78]	Medicine	University specific (VIC)	Longitudinal study (AHPRA, data from Monash University, and MSOD database)	Investigate the rate at which medical graduates with greater than or equal to 12 months of rural training in a region, return to practice in that region in their early medical career (less than 10 years post-graduation).	Geographical region in which the graduate was practicing	Location of training was geocoded using MMM defining rural as MMM2-7. Rural origin was defined as having resided for at least 5 years in MMM2-7 since primary school.	n=702; 59% F; not reported (2007-2015)	29%	Of graduates included, 357 (15%) were practicing in rural Australia. Of these 90 (25%) were working in the same rural area as where they had undertaken their training. Longer duration of rural training (RRR 3.37, 1.89-5.98) and completing schooling and training in the same rural region (RRR 4.47, 2.14-9.36) were associated with returning to practice in the same rural region after training.	Early career medical graduates practicing in rural areas are likely to have prior connections to the region, through either their medical training or secondary schooling, or both.
O'Sullivan & McGrail 2020[79]	Medicine	National (AU)	Longitudinal study (MABEL survey)	To examine how different durations, degree of remoteness and number of	Locations of training and current main practice, and	Locations were geocoded using the MMM with rural defined as MMM2-7.	n=6510;42% F; 43 (median) (1948 – onwards)	Not reported.	Working rurally was associated with having participated in rural	Undergraduate medical training should focus on the number of rural undergraduate

				rural undergraduate medical training placements relate to working rurally.	placement length.				undergraduate medical training (OR 1.6, 95% CI 1.3-1.9), with stronger associations for longer durations (>1 year, OR 3.0, 95% CI 2.3-4.0). Rural background (OR 2.6, 95% CI 2.3-3.0) and general practice (OR 2.6, 95% CI 2.2-2.9) were also associated with working rurally.	training experiences to grow the medical workforce in rural areas.
O'Sullivan et al. 2018[80]	Medicine	University specific (VIC)	Longitudinal study (AHPRA, Monash University medical school data)	To explore associations between various duration and settings of rural immersion during a medical degree, and whether graduates work in a rural location.	Graduates' main work location	Work location was geocoded and categorized using MMM with MMM1 as metropolitan and MMM2-7 as rural. Practice outcomes were also explored by geographical distribution using MMM1 'metropolitan', MMM2 'large regional' and MMM3-7 'smaller regional and rural towns'.	n=2412; 55% F; not reported (2008-2016)	87%	Of included graduates, 14% were working in a rural area. Between two and three years of immersion was strongly related to working in a large regional centre, whereas working in a smaller regional or rural town was associated with rural immersion longer the one year.	Findings support the need for a minimum of one year rural immersion for medical students to increase the rural medical workforce supply.
O'Sullivan et al. 2019[81]	Medicine	University specific (VIC)	Longitudinal study (AHPRA, Monash	To evaluate rural work location outcomes of an	Graduates' main work location.	Work location was geocoded and categorized	n=2412; 63% F (ERC with 2-3 years rural	Not reported	ERC and non-ERC groups with equivalent duration	Findings support that ERC is a valuable program

			University medical school data)	Extended Rural Cohort (ERC) program in medical school.		using MMM with MMM1 as metropolitan and MMM2-7 as rural. Practice outcomes were also explored by geographical distribution using MMM1 'metropolitan', MMM2 'large regional' and MMM3-7 'smaller regional and rural towns'.	training), 61% F (non-ERC students with 2-3 years rural training), 53% F (non-ERC students with less than 2 years training); not reported (2008-2016)		of rural clinical training had similar odds of working in large regional centres (RRR 3.94-5.58). The ERC group was not associated with working in smaller towns, however, the non-ERC group with equivalent rural training was.	which attracts rural background students and increases the uptake of rural practice in early career medical graduates.
Tchia et al. 2019[85]	Dentistry	University specific multiple (QLD, SA, NSW, VIC)	Cross-sectional study (online survey)	To investigate the practice location of Australian dental graduates who completed their degrees from three rurally focused and three metropolitan dental programs in 2015.	University attended, suburb of main practice during their postgraduate year.	Location was categorized using the Modified Monash Model (MMM); MMM1 were classified as "metropolitan", MMM2-3 as "regional, and MMM4-6 as "rural/remote".	n=46; not reported; not reported (2015)	Not reported.	Of participants, n=20 (42%) worked in a metropolitan location, n=14 (30%) worked in large and medium-large regional locations, and n=12 (26% worked in remote or small-to-medium regional areas. Graduates from rural universities were statistically more likely to practice in MMM2-6 in their graduate year, compared to graduates from metropolitan universities.	Dental students attending rural universities are more likely to work in regional and rural areas in their graduate year.

Walker et al. 2021[82]	Medicine	University specific (SA)	Cohort study (AHPRA and Flinders University data)	To compare the graduates of Flinders University medical program who completed training in a metropolitan hospital with those who participated in the Parallel Rural Community Curriculum Rural Clinical School in rural towns.	Registration type, specialty and current location of practice in 2017.	Postcodes of current locations of practice were transformed to MMM with MM2-7 understood to be non-metropolitan areas.	n=1121; Flinders metro 50% F: Flinders rural 59% F; not reported (1999-2012)	89%	In 2017, more than one third of PRCC graduates were working in non-metropolitan areas (MM2-7) compared with 20% of metropolitan graduates (OR 2.2; 95% CI 1.6,3, p<0.001).	Graduates of the PRCC are more likely to work in rural areas, supporting this as a strategy to address the maldistribution of the medical workforce in rural areas.
Cheek et al. 2017[83]	Medicine	University specific (TAS)	Mixed methods with cohort study (University records, interviews and AHPRA 2016 work locations)	To understand the career intentions and work locations of international fee-paying medical students from a medical program in Tasmania, Australia.	Work locations and graduate intentions and factors contributing to their decisions about work locations.	The MMM was used. Locations MM5-MM7 were considered areas of medical workforce shortage. Graduate 2016 work location postcode was matched to MM using Doctor Connect.	n=189; not reported; not reported (2000-2015)	72%	Of graduates, 72.4% were working in Australia, with most (67.7%) working in MM1. A higher proportion of recent graduates (PGY1/2) were working in Tasmania, compared to PGY3+ who were more likely to work in major cities.	International fee-paying medical students make an important contribution to the Australian medical workforce, with a high proportion remaining in the Tasmanian workforce in the first few years following graduation. After this, graduates moved to metropolitan centres.
Drovandi et al. 2020[86]	Pharmacy	University specific (QLD)	Cross-sectional study (JCU Alumni records, AHPRA,	To investigate whether the regional	Practice locations of graduates	Doctor Connect website used to identify MMM classification for	n=847; not reported; not reported (2002-2018)	87%	JCU pharmacy graduates were significantly more likely to practice in	Regionally focused programs attract students from rural backgrounds. The

			National Health Workforce Dataset, IRSAD)	pharmacy school at James Cook University in North Queensland is providing graduates geared to address the pharmaceutical needs of the state's regional, rural and remote communities.		the largest town lying within the LGA.			QLD communities with lower IRSAD scores and higher MMM classifications than other Australian pharmacy program graduates.	program coupled with rural background, increases the likelihood of rural and remote practice and practice in locations with greater socio-economic disadvantage.
May, Brown & Burrows 2018[84]	Medicine	University specific (NSW)	Retrospective cross-sectional study (University data and AHPRA)	To investigate the influence of an extended RCS placement and rural origin on the rural principal place of practice of the first 3 graduate cohorts (2012–2014) from a Joint Medical Program offered by two universities based in northern New South Wales.	Principal place of practice	Practice locations were classified into district of workforce shortage using MMM where category MMM1-2 were metropolitan and regional locations and MMM3-4 represents large to medium regional/rural populations.	n=426; 54% F; 32% 25 years≤ at graduation (2012-2014)	98%	Participation in an extended RCS placement (odds ratio (OR), 6.075, 95% confidence interval (CI) 2.716–13.591), rural background (OR 3.613, 95% CI 1.752–7.450) and being 25 years or older at completion of a medical degree (OR 2.550, 95% CI 1.252–5.194) were all associated with practicing rural.	Strategies to improve the distribution of the rural workforce should include promoting rural placements, in addition to attracting students from a rural background.
**Multiple geographical classification systems or socio-economic classifications used**									
Gurbuxani, Kruger & Tennant 2012[28]	Dentistry	University specific (WA)	Retrospective cohort study (data linkage: Australian Dental Association Directory, Western	To examine the geographic distribution of practice locations of recently graduated dentists from the	Practice location of graduate dentists.	Postcodes were assigned to practice locations. The Accessibility/Remoteness Index for Australia (ARIA)	n=207, not reported, not reported (2004-2009)	91%	Of graduates included, 83% practiced in WA and 17% practiced in other states or overseas. 51% of graduates practiced	Most dentist graduates practice in high socio-economic areas and in accessible areas. Strategies to recruit and retain graduate

			Australian Government Gazette, Yellow Pages Directory, social network sites and email contact confirmed directly with practitioner)	University of Western Australia.		and Socio-Economic Indexes for Areas (SEIFA) were used to classify postcodes. Location data was stratified according to ARIA categories: 1: Highly accessible areas 2: Accessible 3: Moderately accessible 4: Remote 5: Very remote.			in high SES areas, with 11.5% in low SES areas. 78% of practice locations were in ARIA 1 (high accessible areas), with 13% in ARIA 2 (accessible), 5.8% in ARIA 3 (moderately accessibly), 1.9% in ARIA 4 (remote) and 0.5% in ARIA 5 (very remote).	dentists in rural areas is required.
McGirr et al. 2019[68]	Medicine	National (AU)	Cross-sectional study (Rural Clinical School (RCS) de-identified data and AHPRA)	To determine the association between rural location of practice in 2017 and extended rural clinical placement and rural background for 2011 medical graduates from Rural Clinical Schools across Australia.	Rural background, participation in an extended rural clinical placement and whether students were working in a rural or metropolitan area.	Rural background: living in ASGC-RA 2-5 area for at least 5 years since beginning primary school. Place of practice: classified as rural and metropolitan using the ASGC and MMM - rurality was RA2-5 and MMM3-7.	n=1695; 48.8-63.8% F; not reported (2011)	Not reported.	16.6% of 2011 graduates were working in rural areas in 2017 using the ASGC criteria. When using the MMM, 8.3% of graduates were working in rural areas in 2017. Students who attended a RCS were 1.5 times more likely to be in rural practice using the ASGC criteria, and 2.6 times more likely using the MMM criteria.	Students of an extended RCS had an increased likelihood of working rurally following graduation.

Shires et al. 2015[30]	Medicine	University specific (TAS)	Cohort study (AHPRA)	To quantify the proportion of medical graduates who worked in an Australian regional or remote location, or in the regional cities and smaller towns within Tasmania.	Practice location in 2014.	Locations of practice were determined using the postcode listed in the AHPRA database. These postcodes were mapped against ASGC-RA and the 2011 Census population data for Tasmania to define MMM classifications.	n=869; 51% F; 24 (median) (2002-2013)	89%	Students who had spent a year at UTAS RCS were 5 times more likely to be working in RA3 to RA5 than those who had not. Using the MMM, UTAS RCS graduates were 9 times more likely (OR 9.0, 95%CI 4.7–17.2) to be working in the regional cities and smaller towns of Tasmania.	Training medical students in rural areas produces graduates who practice rurally.
**Other geographical or socio-economic classification used**										
Bentley et al. 2019[87]	Medicine	University specific (SA)	Cross-sectional study (survey)	To consider rural practice self-efficacy and its influence on rural career choice by doctors.	Rural self-efficacy; current and intended location of practice in small rural communities (<25 000 people).	Self-reported participant categorization of current practice location as: capital city, major urban centre (>100 000), regional city or large town (>25 000 - 100 000), small town (10 000 - 25 000) or smaller community (<10 000).	n=102; 56.9% F; age not reported (students 1997-2015)	41%	Of participants, 28.5% were working in communities of <25 000 people at the time of completing the survey. Doctors working in smaller towns (<25 000) and small communities (<10 000) had higher rural practice self-efficacy scores.	Implementing targeted strategies to develop an individual's rural practice self-efficacy may improve the retention and recruitment of doctors to small rural communities.
Playford, Wheatland & Larson 2010[88]	Nursing	University specific multiple (WA)	Longitudinal cohort study (survey)	To determine the undergraduate education most related to rural recruitment for	Graduate location.	Location was coded as urban or rural. Rural was classified according to the	Rural cohort: n=49, not reported; not reported. Urban cohort: n=100, not	Rural cohort: 87%; Urban cohort: 78%	Of the rural cohort, 43% were in rural nursing positions, compared to the urban cohort where	Rurally located education may increase the likelihood of nursing graduates

				nursing graduates in Western Australia.		University of Western Australia definition as towns more than 100 kilometers from the city post office.	reported; not reported (2000-2004)		25% were in rural nursing positions.	working in a rural area.
Puddey, Playford & Mercer 2017[27]	Medicine	University specific (WA)	Longitudinal cohort study (University of Western Australia data, AHPRA)	To examine whether medical students from socio-economic disadvantaged backgrounds will practice in areas of increased socio-economic disadvantage.	Practice location, rural origin, Index of Relative Socio-economic Advantage and Disadvantage (IRSAD)	Postcode of current practice address was dichotomized into top 2 deciles of IRSAD versus the bottom 8 deciles.	n=2829; 48% F; 24.6 years (mean age at graduation) (1980-2011)	90%	Those categorized in the lower 8 socio-economic deciles at medical school entry had increased odds of currently practicing in the lower 8 socio-economic deciles following five years after graduation (OR 2.05, 95% CI 1.71,2.45, p<0.001), even after adjusting for potential confounders.	Implementing strategies to target participation of students from diverse socio-educational backgrounds in medical school, may increase the distribution of the medical workforce across socio-economic areas.
Woolley, Hogenbirk & Strasser 2020[26]	Medicine	University specific multiple including international (QLD and Canada)	Cross-sectional study (online survey)	To identify commonalities between one regionally based medical school in Australia and one in Canada regarding the association between postgraduate training location	Practice location in 2018 and completion of postgraduate training.	For JCU graduates, current practice location was dichotomized into whether or not the town was in northern Australia (from Rockhampton northwards and westwards to the Northern	Graduates who had completed PGY5 by 2018: n=197; not reported; not reported (2005-2013)	26%	Of respondents, 38% of JCU graduates who had completed their Fellowships, were practicing in northern Australia in 2018 (JCU medical school's reference area).	The study supports the need to have full specialty training programs located in rural and regional clinical settings to increase medical graduates in these areas.

				and a doctor's practice location.		Territory and to Carnarvon in Western Australia).				
Hays et al. 2017[89]	Medicine	University specific (TAS)	Retrospective cohort study (AHPRA)	To report the workforce outcomes of the first 42 graduating cohorts from the University of Tasmania medical school.	Current practice location, specialty and registration details.	Locations aggregated by state/territory and international.	n=1707; 43% F; 42 years at graduation (mean) (1971-2011)	85%	Of graduates with place of practice data available (n=1707), the largest proportion were practicing in Tasmania (35.6%), followed by Victoria (15.3%) and NSW (11.8%).	The Tasmanian medical program is understood to provide a substantial contribution to the Tasmania workforce.
Woolley, Gupta & Murray 2016[90]	Medicine	University specific (QLD)	Longitudinal cohort study (AHPRA, JCU data)	To describe the different outcomes of the strategies used at the JCU medical school to improve the recruitment and retention of medical graduates in northern Australian towns.	Practice location	Internship and current practice location were categorized into the associated town or Hospital and Health Services District (HSD). HSDs were based on the boundaries map obtained from the Queensland Health website.	n=742; not reported; not reported (2005-2013)	97%	The strongest predictor of JCU medical graduates undertaking their internship in Townsville, Cairns, Mackay or Darwin hospitals was attending the JCU clinical school in that location (p<0.001, prevalence odds ratios (POR)=7.1; p<0.001, POR=11.5; p<0.001, POR=19.4; p<0.001, POR=85.7; respectively).	Study supports the effectiveness of improving rural medical workforce through targeted selection of rural background students.
**No geographical or socio-economic classification system reported**									
Forster et al. 2013[31]	Medicine	University specific (NSW)	Retrospective cohort study (online survey)	To understand the influence of the number of years spent at an Australian RCS	Current practice location, preferred current practice	Locations were based on five regional categories including inner	n=214; 48% F; not reported (2003-2010)	68%	Of respondents, 26% were currently working in rural areas, with 50% expressing	RCS experience greater than a year is likely to be associated with medical graduates'

				on graduate current, preferred current and intended location for rural workforce practice.	location and their intended graduate practice location.	urban, outer metropolitan, regional, rural and remote areas, dichotomized into urban: inner urban and outer metropolitan, and rural: regional, rural and remote areas but no categorization system was reported to define these categories.			preference to be currently working in a rural area, and 67% intending to work in a rural area after completion of medical training. Graduates from a non-rural background who spent three years at a rural campus were more likely to take up rural practice when compared to those who spent one year at a rural campus (OR 8.4, 95% CI 2.1,33.5, p=0.002).	practicing rurally and intending to work in a rural area.
Playford et al. 2013[32]	Medicine	University specific (WA)	Longitudinal cohort study (AHPRA and follow up of graduates)	To compare rural location identified through the AHPRA registry with location obtained through labour intensive personal contact.	Percentage of location matches between the two contact methods.	Not reported.	n=330; not reported; not reported (participated in RCS 2002-2009)	Not reported.	There was 80% agreement for principal suburb, 92% agreement for principal city and 94% agreement for principal state between RCSWA personal contact and the AHPRA registry.	AHPRA data matched RCSWA alumni data only for graduates in full-time rural work.

1.
